# The effect of health on social capital; a longitudinal observation study of the UK

**DOI:** 10.1186/s12889-020-08577-w

**Published:** 2020-04-07

**Authors:** Paul Downward, Simona Rasciute, Harish Kumar

**Affiliations:** 1 School of Sport, Exercise and Health Sciences, Loughborough University, Ashby Road, Loughborough, LE11 3TU UK; 2 School of Business and Economics, Loughborough University, Ashby Road, Loughborough, LE11 3TU UK

**Keywords:** Social capital, Bonding, Bridging, General health, Mental health, Gender, Aging

## Abstract

**Background:**

UK health policy increasingly focusses on health as an asset. This represents a shift of focus away from specific risk factors towards the more holistic capacity by which integrated care assets in the community support improvements in both health and the wider flourishing of individuals. Though the social determinants of health are well known, relatively little research has focussed on the impact of an individual’s health on their social outcomes. This research investigates how improved health can deliver a social return through the development of social capital.

**Methods:**

An observational study is undertaken on 25 years of longitudinal data, from 1991, drawn from the harmonised British Household Panel Survey (BHPS) and Understanding Society Survey (USS). Fixed effects instrumental variable panel data regression analysis is undertaken on individuals. The number of memberships of social organisations, as a measure of structural social capital, is regressed on subjectively measured general health and GHQ12 (Likert) scores. Distinction is drawn between males and females.

**Results:**

Improved general health increases social capital though differences exist between males and females. Interaction effects, that identify the impacts of health for different age groups, reveal that the effect of increased health on social capital is enhanced for males as they age. However, in the case of females increases in general health increase social capital only in connection with their age group. In contrast mental illness generally reduces social capital for males and females, and these effects are reduced through aging.

**Conclusions:**

Investing in health as an asset can improve the social outcomes of individuals. Increasing the outcomes requires tailoring integrated care systems to ensure that opportunities for social engagement are available to individuals and reflect age groups. Targeting improvements in mental health is required, particularly for younger age groups, to promote social capital. The results suggest the importance of ensuring that opportunity for engagement in social and civic organisation be linked to general and mental health care support.

## Background

The social determinants of health (SDH) such as levels of education, age, ethnicity, social and occupational status, and income have been extensively researched for both physical and mental health outcomes [1–3]. Social capital has also been identified as a social determinant of health [4]. There is debate over the nature and characterisation of social capital. Economic accounts identify social capital as an individual’s accrual of social characteristics [5]. Sociology and political-science identify social capital as a feature of groups, for example social classes [6], or based in both groups and individuals and reflected in civic participation, social norms, and trust [7, 8]. Regardless, a general feature of the social capital literature is that when social capital declines, ‘bad’ social outcomes occur, whatever outcomes might be examined [9] and this result is shared in the SDH literature [10, 11].

The SDH literature has contributed to this general insight recognising that social capital has structural, relational and cognitive features. All of these refer to different dimensions by which social connectedness can be facilitated through social proximity. Structural social capital relates to the ‘quantity’ of social capital possessed by individuals and it has long been linked to the number of associational activities undertaken, as a measure of the scale and extensiveness of more formal connections. These can include voluntary associations as well as civic and work-related associations [5, 12–16]. Relational and cognitive concepts of social capital relate to its ‘quality’, associated with subjective feelings, perceptions of the support and reciprocity of, and trust placed in other individuals [17, 18]. The mechanisms by which social capital promotes health are argued to rest in the moral, knowledge and resource support that is available to individuals to meet both mental and physical health challenges [19].

Understanding the SDH and the role of social capital is clearly important for health policy both in understanding and addressing health inequalities [20] and the incidence of non-communicable diseases [21]. However, current UK health policy, captured in the latest NHS Long-term plan [22] drawing on the World Health Organisation [23], now also stresses the role of health as an asset [24]. This recognises that as part of an integrated care system, intersectoral policy co-operation is necessary not only to encourage other policy domains to become accountable for health, but also to support health as important in the promotion of a more inclusive and productive society [25]. Treating health as an asset, therefore, implies a shift away from a health policy focus on specific risk factors and towards a more holistic perspective on the promotion of health and flourishing more generally [26]. Moreover, this identifies the policy importance of examining the impact of health on social capital, as an important social outcome.

There is recognition in the SDH literature of the need to focus on the impact of health on social capital. This has arisen with concerns over the potential endogeneity and direction of causality between social capital and health [19, 27]. For example, greater mental well-being might be needed to increase social engagement as much as social engagement can improve mental well-being [28]. Moreover, despite arguments that empirical exploration of the direction of the relationship is not well developed [29], some support for the causal impact of health on social capital has been identified. Improvements in mental health have been shown to be connected to greater civic engagement for men [28]; declines in self-rated health with reductions in levels of trust in others; both improved mental and physical health with enhanced social networks [30]; greater physical health with more engagement in social activities [31]; and, declines in physical health linked to ageing with less engagement in social activities [32].

Significant characteristics of this literature are that it recognises a need to address causality by making use of longitudinal data [19, 27], employing fixed effects to control for time invariant heterogeneity across individuals [27, 28, 33], and relevant lagged or instrumental variable estimation [17, 31]. Consequently, this research draws upon longitudinal data, and makes use of instrumental-variable fixed effects panel estimation to explore the impacts of health on social capital in the UK. Moreover, the research explores the differences connected with general and mental health, and also males and females, the latter of which has been identified in the literature as especially important [34]. The analysis controls for confounders that are typically cited in the literature and which have the possibility of time variation. A fixed effect (dummy) variable is included to control for the use of two surveys.

## Methods

The aim of the study is to examine the causal influence of general and mental health on social capital. A longitudinal observational design is employed based upon 25 years of data, from the harmonised British Household Panel Survey (BHPS) and Understanding Society Survey (USS) [35, 36]. The BHPS is a longitudinal social survey of households and individuals living in the UK. It began in 1991 and lasted until 2008, when it was superseded by, and absorbed into the USS, which has much larger samples in its annual waves [35].

A structural measure of social capital is adopted which measures the number of memberships of individuals in social, civic and community groups [5, 12]. These include: a political party, a trade union, an environmental group, a parents association, a tenants or residents association, a religious group, a voluntary service group, a pensioners organisation, a scout/guides organisation, a professional organisation, another community group, a social group, a sports club, the women’s institute, a women’s group and ‘other’ organisations. Memberships of each of these are identified as a yes or no response as a binary indicator. These were then summed to obtain the total number of associations that individuals are members of [37].

Two measures of health are employed. The first is a general health variable that measures an individual’s subjective view of their overall health, and has been identified as broadly, but not exclusively, a measure of physical health [17, 38]. This is measured on a five-point Likert scale in both the BHPS and USS. However, the response categories are different. In the BHPS general health has following dimensions: excellent, good, fair, poor and very poor. In the USS these are: excellent, very good, good, fair and poor. An approximate common measure was created based on the sample proportions of four values as: excellent, good, fair, very poor. This scale combined the ‘very poor’ and ‘poor’ scales in BHPS and the ‘very good’ and ‘good’ scales of USS data to match. Although this introduces approximation in the variation in the measure it allowed for analysis of the complete time-series of data. To improve the precision of the analysis because of this, and because in large cross-sections of data, as with each wave in this dataset, variations in the data can be influenced by different sub-populations or groupings, heteroscedasticity was controlled for by basing inference on robust standard errors [39]. The second measure of health is based on the 12 item General Health Question (GHQ12) which measures mental health [40]. This is consistently measured across the BHPS and USS data, and the Likert version, that is the total value of the scores of its items, is adopted in this study [41–43].

Analysis was undertaken using the instrumental variable fixed effects panel data estimator from Stata version 16 [10]. This estimator is used for two main reasons. Because the data is longitudinal (i.e. panel) the use of fixed effects controls for unmeasured personal characteristics that may determine both health status and social capital but are assumed to be constant over time [28]. Instrumental variables are then used to identify the causal impacts of the health measures on social capital. Without these only an association between the variables would be identified, which includes the possibility that social capital influences health. The instrumental variables employed in the analysis included the lagged value of health as well as dummy variables for the countries in the UK (England, Scotland and Wales compared to Northern Ireland and the Channel Isles). The former measures the health status that has occurred prior to the measurement of the social capital membership status. The country dummies, it is assumed, capture elements of the national variation in supply of health services [44]. Importantly, the adequacy of the instruments is assessed statistically. F-tests are undertaken to assess the joint relevance of the instruments. These are based on a regression of the measures of health on the instruments and other confounding variables. The Hansen-Sargan test is then undertaken to examine the independence of the instrumental variables from the unmeasured errors of the instrumental variable regression and hence their validity [45]. The health, social capital, instrumental variables and confounding variables included in the analysis are described in Table 1, with descriptive statistics given for each measure of health. Analysis was also undertaken allowing health to interact with different age groups to explore age-specific health effects on social capital. This is because of the obvious link between ageing and health [46]. The age groups of 16 to 29 years; 30 to 64 years; and, 65 years plus were adopted to both echo WHO guidelines on health and physical activity but also recognising that a lot of civic and social engagement, for example through volunteering, tends to take place in middle age rather than younger or very old age groups [47]. The use of age groups in the interactions distinguishes the effect of the constant accrual of age from how health at a particular stage of life affects social capital. Research also shows that this tends to differ for both males and females, with males having more discretion over their leisure-time than females to volunteer in the UK [48], but that females are more likely to volunteer as they get older [49]. Consequently, it is hypothesised that improvements in general and mental health will be connected with increases in structural social capital, and that this is more likely with older age groups. In addition, it is likely that the effects will be stronger for males generally, but that the effects will become more pronounced for females over aging groups.

**Table 1:** Variable Descriptions and Characteristics

		General Health		GHQ12	
Variable	Description	Mean	Std dev.	Mean	Std. Dev.
Orgnumber	Number of memberships of organisations	0.954	1.156	0.967	1.163
General Health	General Health scale (1 - very poor to 4 - Excellent)	2.880	0.823		
GHQ12	GHQ12 Likert scale (0 to 36)			11.083	5.412
Age	Age in years	47.430	18.091	47.156	17.964
Age0	Aged between 16 and 29 years	0.190	0.392	0.192	0.394
Age1	Aged between 30 and 64 years	0.606	0.489	0.609	0.488
Age2	Aged 65 years and above	0.204	0.403	0.199	0.399
BHPS	Data from the BHPS survey compared to the USS	0.584	0.493	0.597	0.491
Sex	Gender (1 - male, 0 female)	0.447	0.497	0.447	0.497
Couple	Married or a couple versus other marital status (1- yes, 0- no)	0.555	0.497	0.558	0.497
Higher Education	Has a degree or equivalent (1- yes, 0- no)	0.173	0.378	0.172	0.378
Child 0–2	Number of children aged 0 to 2 years	0.079	0.290	0.079	0.290
Child 3–4	Number of children aged 3 to 4 years	0.069	0.265	0.069	0.266
Child 5–11	Number of children aged 5 to 11 years	0.248	0.588	0.250	0.591
Child 12–15	Number of children aged 12 to 15 years	0.160	0.437	0.161	0.437
Real Family Income	Real gross monthly household income (£)	1652.50	1602.82	1654.99	1604.42
Self employed	Self employed (1- yes, 0- no)	0.073	0.260	0.073	0.260
Employed	Employed full time (1- yes, 0- no)	0.502	0.500	0.508	0.500
Unemployed	Unemployed (1- yes, 0- no)	0.038	0.192	0.038	0.191
Retired	Retired (1- yes, 0- no)	0.227	0.419	0.222	0.415
Maternity leave	On maternity leave (1- yes, 0- no)	0.005	0.068	0.005	0.068
Family care	Caring for the family (1- yes, 0- no)	0.067	0.250	0.067	0.250
Full-time study	In full-time study (1- yes, 0- no)	0.045	0.207	0.045	0.206
Long-term sick	On long-term sick leave (1- yes, 0- no)	0.037	0.190	0.036	0.187
England	Respondent is from England (1- yes, 0- no)	0.692	0.462	0.710	0.454
Scotland	Respondent is from Scotland (1- yes, 0- no)	0.129	0.335	0.126	0.332
Wales	Respondent is from Wales (1- yes, 0- no)	0.107	0.309	0.103	0.304
n		150, 298		153, 382	

## Results

Table 2 provides the average number of organisations that individuals are members of by age groups and sex for the two samples over which general health and mental health are analysed. Table 3 provides the pairwise correlations between the number of organisations, age, sex, and the health measures (the lower left diagonal for general health and the upper right diagonal for mental health). Both tables suggest that structural social capital is positively associated with improved health, older age groups and being male. However, to formally test the hypotheses above, accounting for confounding influences, Tables 4 and 5 present the regression results. The tests for the relevance and validity of the instrumental variables are reported at the bottom of each table. The statistically significant F-statistics and insignificant Sargan-Hansen statistics indicate evidence in favour of the relevance and validity of the instruments. Consequently, some causal evidence for the impact of health on social capital is identified. Consistent with the hypotheses above, Table 4 presents evidence in favour of increased general health and the accrual of social capital as the coefficient estimates are positive. The size of the effect is larger for males. Likewise, the results for GHQ12 reveal that reductions in mental health, measured by higher GHQ12 scores, contributes to a reduction in social capital for females, as given by the statistically significant negative coefficient, but not males. Table 5 presents the results that also include the health and age-group interactions. These results indicate that when controlling for the stage of life in the analysis by including interaction effects, increased general health remains a factor that leads to the accrual of social capital for males as indicated by the positive coefficient sign, and this accrual is reinforced across age groups as the coefficients on the interaction terms are positive. In contrast, for females, general health is shown to improve social capital when linked to their older life-stages only. This is indicated by the positive coefficients being significant only for the interaction effects. The relationship between social capital and general health is, thus, more life-stage dependent for females as also hypothesised above. In the case of mental health, the results suggest that increased GHQ12 scores, associated with declines in mental health, reduce the social capital of both males and females. However, the interaction effects show that relative to a younger life-stage the impact of reduced mental health on reductions in social capital is less in older life-stages. This is because the coefficient values are positive compared to the negative value of the coefficient on GHQ12 not interacted with age groups. The size of the interaction effects is also greater for females, which is consistent with the hypotheses above. Consequently, overall, whilst ageing is generally shown to reduce the social capital for individuals the results indicate that life-stages are an important intermediary in health both for developing social capital and retaining it.

**Table 2:** Mean number of organisations by Age group and gender

	General Health		GHQ12	
Variable	Mean	Std dev.	Mean	Std. Dev.
Age0	0.619	0.005	0.625	0.005
Age1	1.026	0.004	1.039	0.004
Age2	1.055	0.007	1.077	0.007
Female	0.899	0.004	0.899	0.004
Male	1.023	0.004	1.023	0.004
n	150,298		153,382

**Table 3:** Correlations of Organisational Memberships, Age, Health, Sex

	Orgnumber	Age	Sex	GHQ12
Orgnumber	1	0.1226***	0.0546***	−0.0565***
Age	0.1176***	1	− 0.0079***	0.0028
Sex	0.053***	− 0.0098***	1	− 0.1191***
General Health	0.1026***	− 0.1975***	0.0507***	1

^*^p < 0.10, ^**^p < 0.05, ^***^p < 0.01

**Table 4:** Instrumental Variable Fixed Effect Panel Regression Estimates

	All	Male	Female	All	Male	Female
General health	0.0895^***^	0.117^***^	0.0606^**^			
	(4.04)	(3.49)	(2.06)			
GHQ12				− 0.00968^***^	− 0.00486	− 0.0122^**^
				(−2.61)	(− 0.86)	(−2.49)
Age	− 0.0105^***^	− 0.0135^***^	− 0.00814^***^	−0.0111^***^	− 0.0147^***^	− 0.00828^***^
	(−10.98)	(−9.22)	(−6.41)	(− 11.93)	(− 10.57)	(−6.60)
BHPS	− 0.126^***^	−0.121^***^	− 0.130^***^	−0.123^***^	− 0.122^***^	−0.124^***^
	(− 10.68)	(−6.80)	(−8.27)	(− 10.53)	(−6.93)	(−7.91)
Couple	0.0632^***^	0.0619^***^	0.0724^***^	0.0591^***^	0.0532^***^	0.0710^***^
	(4.98)	(3.14)	(4.38)	(4.68)	(2.76)	(4.28)
Higher Education	0.193^***^	0.113^**^	0.247^***^	0.177^***^	0.0899^**^	0.235^***^
	(6.22)	(2.56)	(5.84)	(5.76)	(2.05)	(5.63)
Child 0–2	−0.0575^***^	−0.0752^***^	−0.0434^***^	−0.0575^***^	− 0.0782^***^	−0.0405^***^
	(−5.64)	(−4.87)	(−3.20)	(−5.81)	(−5.24)	(−3.06)
Child 3–4	−0.0239^**^	−0.0376^**^	− 0.0107	−0.0225^**^	− 0.0394^**^	−0.00697
	(−2.27)	(−2.30)	(−0.78)	(−2.18)	(− 2.46)	(− 0.51)
Child 5–11	0.0649^***^	0.0277^***^	0.0943^***^	0.0643^***^	0.0301^***^	0.0911^***^
	(9.22)	(2.58)	(10.14)	(9.31)	(2.87)	(9.97)
Child 12–15	0.0470^***^	0.0427^***^	0.0519^***^	0.0493^***^	0.0424^***^	0.0558^***^
	(5.88)	(3.46)	(4.94)	(6.29)	(3.55)	(5.39)
Real Family Income	0.0000197^***^	0.0000194^***^	0.0000226^***^	0.0000201^***^	0.0000186^***^	0.0000252^***^
	(6.36)	(4.99)	(4.39)	(6.53)	(4.82)	(4.93)
Self employed	−0.0294	− 0.0125	− 0.0154	− 0.0333	− 0.0167	− 0.0269
	(− 0.82)	(− 0.24)	(− 0.31)	(− 0.95)	(−0.33)	(− 0.54)
Employed	0.0473	0.0992^**^	0.0120	0.0431	0.0904^*^	0.0101
	(1.45)	(2.00)	(0.28)	(1.36)	(1.91)	(0.24)
Unemployed	−0.0435	0.00134	− 0.0809^*^	−0.0345	− 0.00631	−0.0597
	(−1.27)	(0.03)	(−1.76)	(−1.02)	(−0.13)	(−1.29)
Retired	−0.0667^*^	−0.0436	− 0.0831^*^	−0.0864^**^	− 0.0805	− 0.0895^**^
	(− 1.91)	(− 0.80)	(− 1.83)	(−2.52)	(− 1.54)	(− 1.97)
Maternity leave	− 0.0210		− 0.0536	− 0.0373		− 0.0697
	(− 0.44)		(− 0.97)	(− 0.79)		(− 1.27)
Family care	−0.0147	− 0.0580	− 0.0354	− 0.0138	− 0.0405	−0.0277
	(−0.43)	(−0.82)	(− 0.81)	(− 0.41)	(−0.61)	(− 0.63)
Full-time study	0.0809^**^	0.135^**^	0.0473	0.0675^*^	0.106^*^	0.0448
	(2.14)	(2.31)	(0.95)	(1.84)	(1.90)	(0.92)
Long-term sick	−0.0535	−0.0289	−0.0746	− 0.0624^*^	−0.0764	− 0.0554
	(−1.45)	(−0.51)	(− 1.55)	(− 1.70)	(− 1.40)	(− 1.12)
Constant	1.147^***^	1.241^***^	1.073^***^	1.560^***^	1.733^***^	1.401^***^
	(12.05)	(8.40)	(8.66)	(23.48)	(17.93)	(15.39)
*n*	150,298	67,144	83,154	153,382	68,525	84,857
Sargan-Hansen χ^2^(3)	4.870	5.081	3.511	4.121	5.266	2.894
*First-stage*						
F(4, 34,423)	558.74^***^					
F(4, 15,243)		248.59^***^				
F(4, 19,176)			307.87^***^			
F(4, 34,499)				295.55^***^		
F(4, 15,270)					132.36^***^	
F(4, 19,225)						165.50^***^

**Table 5:** Instrumental Variable Fixed Effect Panel Estimates (including age-group interaction effects)

	All	Male	Female	All	Male	Female
General health	0.0423^*^	0.0824^**^	0.00360			
	(1.78)	(2.29)	(0.11)			
General health*Age1	0.0390^***^	0.0279^***^	0.0490^***^			
	(5.74)	(2.77)	(5.29)			
General health*Age2	0.0793^***^	0.0550^***^	0.102^***^			
	(6.94)	(3.14)	(6.68)			
GHQ12				−0.0202^***^	−0.0136^*^	− 0.0226^***^
				(−4.58)	(−1.95)	(−3.96)
GHQ12*Age1				0.0105^***^	0.00894^***^	0.0103^***^
				(5.36)	(2.73)	(4.16)
GHQ12*Age2				0.0125^***^	0.00989^*^	0.0127^***^
				(4.03)	(1.91)	(3.21)
Age	−0.0137^***^	− 0.0157^***^	− 0.0122^***^	− 0.0134^***^	− 0.0164^***^	− 0.0108^***^
	(− 12.86)	(−9.79)	(−8.53)	(− 12.85)	(− 10.57)	(−7.63)
BHPS	− 0.125^***^	− 0.122^***^	− 0.127^***^	−0.125^***^	− 0.124^***^	−0.125^***^
	(− 10.65)	(−6.86)	(−8.12)	(− 10.67)	(−7.02)	(− 8.00)
Couple	0.0524^***^	0.0527^***^	0.0607^***^	0.0487^***^	0.0432^**^	0.0611^***^
	(4.06)	(2.62)	(3.61)	(3.81)	(2.19)	(3.64)
Higher Education	0.191^***^	0.111^**^	0.246^***^	0.170^***^	0.0846^*^	0.229^***^
	(6.18)	(2.52)	(5.84)	(5.57)	(1.93)	(5.49)
Child 0–2	−0.0679^***^	−0.0832^***^	−0.0557^***^	−0.0640^***^	− 0.0837^***^	−0.0470^***^
	(−6.70)	(−5.40)	(−4.14)	(− 6.51)	(−5.62)	(−3.58)
Child 3–4	−0.0366^***^	−0.0480^***^	− 0.0252^*^	−0.0318^***^	− 0.0474^***^	−0.0162
	(− 3.49)	(−2.92)	(−1.84)	(− 3.08)	(−2.95)	(− 1.20)
Child 5–11	0.0522^***^	0.0197^*^	0.0768^***^	0.0529^***^	0.0226^**^	0.0779^***^
	(7.26)	(1.83)	(7.94)	(7.45)	(2.13)	(8.15)
Child 12–15	0.0374^***^	0.0383^***^	0.0368^***^	0.0396^***^	0.0374^***^	0.0436^***^
	(4.62)	(3.10)	(3.40)	(4.95)	(3.10)	(4.07)
Real Family Income	0.0000185^***^	0.0000184^***^	0.0000212^***^	0.0000191^***^	0.0000178^***^	0.0000238^***^
	(5.97)	(4.74)	(4.12)	(6.18)	(4.61)	(4.63)
Self employed	−0.0262	−0.00907	−0.0152	−0.0346	−0.0197	−0.0261
	(−0.73)	(−0.17)	(− 0.30)	(− 0.98)	(−0.39)	(− 0.52)
Employed	0.0498	0.103^**^	0.0124	0.0421	0.0885^*^	0.0101
	(1.54)	(2.08)	(0.29)	(1.33)	(1.88)	(0.24)
Unemployed	−0.0407	0.00570	− 0.0822^*^	−0.0295	− 0.00168	−0.0565
	(−1.20)	(0.11)	(−1.79)	(−0.87)	(−0.03)	(−1.21)
Retired	−0.0861^**^	−0.0625	− 0.101^**^	−0.0793^**^	− 0.0756	−0.0803^*^
	(−2.45)	(−1.13)	(−2.22)	(−2.29)	(− 1.40)	(− 1.76)
Maternity leave	−0.0186		−0.0548	−0.0367		−0.0694
	(−0.39)		(−0.99)	(−0.78)		(−1.25)
Family care	−0.0140	−0.0529	− 0.0362	−0.00906	− 0.0399	−0.0226
	(−0.41)	(−0.75)	(− 0.82)	(− 0.27)	(−0.60)	(− 0.52)
Full-time study	0.0818^**^	0.138^**^	0.0448	0.0643^*^	0.103^*^	0.0416
	(2.17)	(2.38)	(0.90)	(1.75)	(1.85)	(0.85)
Long-term sick	−0.0557	−0.0296	−0.0808^*^	− 0.0565	−0.0730	− 0.0498
	(−1.52)	(−0.53)	(− 1.68)	(− 1.53)	(−1.33)	(− 1.00)
Constant	1.339^***^	1.382^***^	1.308^***^	1.698^***^	1.835^***^	1.551^***^
	(13.40)	(8.97)	(9.97)	(23.22)	(17.10)	(15.50)
n	150,298	67,144	83,154	153,382	68,525	84,857
Sargan-Hansen χ^2^(3)	4.704	5.133	3.595	3.577	5.368	2.665
*First stage*						
F(6, 34,423)	387.91^***^					
	5577.27^***^					
	2165.37^***^					
F(6, 15,243)		169.51^***^				
		2220.16^***^				
		775.10^***^				
F(6, 19,176)			217.73^***^			
			3317.82^***^			
			1370.18^***^			
F(6, 34,499)				212.20^***^		
				2499.68^***^		
				1267.15^***^		
F(6, 15,270)					100.03^***^	
					1069.29^***^	
					509.63^***^	
F(6, 19,225)						116.42^***^
						1454.89^***^
						762.86^***^

*t* statistics in parentheses ^*^p < 0.10, ^**^p < 0.05, ^***^p < 0.01

## Discussion

Current UK health policy increasingly identifies health as an asset, that should play a role in an integrated care system that seeks to promote a more inclusive and productive society that enhances the general flourishing of individuals. The SDH literature also recognises the importance of social capital in the promotion of health but recognises that there is potential endogeneity between health and social outcomes, with some literature, identified above, showing that health can promote social capital. However, the literature also recognises a need to address causality using longitudinal data, fixed effects controls and relevant lagged or instrumental variable estimation. This study has undertaken this task in the UK.

The study adds to the literature in arguing that improvements in general health promotes social capital and facilitates greater capacity for engagement in social and civic organisations as hypothesised. Health can thus facilitate greater social functioning contributing to well-being [50]. In as much that general health includes physical health the results suggest that social capital could increase through a greater capacity to be able to engage in activities. This will be naturally enhanced further if greater relative health exists over later life stages in which ageing can act to reduce engagement [51]. In this regard, the results also indicate that the promotion of social and civic engagement opportunities for younger females linked to health care is especially important. This is because female social and civic engagement is linked to health through their progression through later life-stages. As noted above it is known, for example, that many voluntary engagements are linked to gender [52], but also that older females are more likely to volunteer in the UK [49].

The results also suggest that reduced mental health is an important constraint on younger male and female development of social capital. This is because the impact of reduced social capital from reduced mental health is less in older life-stages. The mechanisms by which this takes place could be that much civic engagement typically takes place in middle and older age groups [47]. Relatively greater social support and organisational recruitment mechanisms in these age groups, and particularly for females, have been shown to counter how reductions in mental health can lead to more social isolation [27]. It should be noted in this regard that because the analysis controls for other confounders that identify the social and economic activity of individuals, as well as employs fixed effects that controls for the influence of person-specific potential propensity to be, for example pro-social or not, the results identify the health specific effects on social capital.

There are limitations of this study. The measurement of general health required approximation rather than being fully consistent across the BHPS and USS components of the harmonised data. Where possible attempts were made to control for this by correcting for heteroscedasticity and including a fixed effect to measure the shift between the BHPS and USS in the harmonised data. The choice of instruments was also constrained by data availability over such a long period and, consequently, were derived from within the data. Though acceptable diagnostic statistics were obtained exploration of more focussed sets of instruments would help, for example drawing upon the physical provision of health care services in the locality of individuals as measures of the actual supply-side of care. Finally, the measure of social capital focusses only on its structural form and there is no identification of the frequency and level or form of engagement that is involved. Addressing all of these limitations is likely to require the use of less longitudinal and potentially primary data and access also to measures of more intensity of engagement in organisations as well as the qualitative and relational dimensions of social capital. These do not exist in the data analysed in this study.

## Conclusions

The analysis shows that investing in health as an asset can improve the social outcomes of individuals, through raising their social capital as well as insulating against the impact that declines in mental health can have on social capital. The results suggest the importance of ensuring that opportunity for engagement in social and civic organisation be linked to general and mental health care support. The analysis informs the case for integrated care systems by indicating that information upon, and opportunities for, social engagement are made available to individuals and are aimed particularly at younger age groups. This is particularly important in the case of the mental health of younger adults.

## Data Availability

The datasets analysed during the current study are available from the UK Data Archive Study Number 6614 https://beta.ukdataservice.ac.uk/datacatalogue/studies/study?id=6614, 10.5255/UKDA-SN-6614-12

## Abbreviations

SDH: Social determinants of health
BHPS: British Household Panel Survey
USS: Understanding society survey
GHQ12: 12 item general health questionnaire
WHO: World Health Organisation
UK: United Kingdom
NHS: National Health Service
